# Influence of a full-spectrum aqueous extract of black cardamom (Amomum subulatum Roxb.) fruits on focus/alertness and executive function: a randomized, double-blind, placebo-and active- controlled, 14-day comparative study in healthy working professionals

**DOI:** 10.3389/fnins.2026.1879347

**Published:** 2026-09-29

**Authors:** Jestin V. Thomas, M. E. Mohan, Syam S. Das, Nithyanandam Allimuthu, Sheena Devasia, P. A. Aneesa, Krishnakumar Illathu Madhavamenon, Baby Chakrapani Pulikkaparambil Sasidharan

**Affiliations:** 1 Leads Clinical Research and Bio-Services Private Limited, Bangalore, India; 2 BGS Global Institute of Medical Sciences, Bangalore, India; 3 R&D Centre, Akay Natural Ingredients Private Limited, Kochi, India; 4 Tamil Nadu Government Multi Super Speciality Hospital, Chennai, India; 5 Centre for Neuroscience, Department of Biotechnology, Cochin University of Science and Technology, Kochi, India; 6 Department of Biochemistry, Sree Sankara College, Kochi, India; 7 Centre of Excellence in Neurodegeneration and Brain Health, Kochi, India

**Keywords:** *Amomum subulatum*, black cardamom, caffeine, CNS vital signs, cognition, executive function

## Abstract

**Background:**

Nootropics enhance cognitive functions, including memory, attention, alertness, mood, and reaction time, through diverse neurobiological mechanisms. Caffeine, one of the most widely consumed nootropics, improves cognitive performance primarily by promoting wakefulness and alertness; however, these effects are generally transient. Consequently, there is growing interest in plant-derived, food-grade nootropics that can provide sustained cognitive benefits with a favorable safety profile. In this context, the nootropic efficacy of a full-spectrum aqueous extract of black cardamom fruits (MA2-24), administered alone and in combination with caffeine, was evaluated in the clinical trial.

**Methods:**

This randomized, double-blind, placebo- and active-controlled, 4-arm parallel group study for 14 days included healthy participants randomized into four groups (*n* = 24/group): placebo (250 mg), MA2-24 (250 mg), caffeine (200 mg), or MA2-24 + caffeine (250 + 200 mg). Cognitive performance was assessed on the 1st, 7th and 14th day at 1-, 3-, 5-, and 8-h post-intervention time points using validated CNS Vital Signs (CNSVS) tasks.

**Results:**

Linear mixed-effects analyses revealed significant effects of treatment, study day, and post-dose assessment time on cognitive performance, with significant interaction effects across outcomes. Supplementation with MA2-24 improved cognitive performance, particularly in measures of attention, processing speed, cognitive flexibility, and executive function, as reflected by increased correct responses, faster reaction times, and fewer errors in the Shifting Attention, Symbol Digit Coding, and Stroop tests. Significant improvements with MA2-24 were observed during the early post-dose period (1–3 h) across multiple cognitive outcomes compared with placebo (*p* < 0.05). Co-administration of MA2-24 with caffeine produced greater improvements at the later post-dose assessments (5–8 h), particularly on Day 14 (*p* < 0.05).

**Conclusion:**

CNSVS tests demonstrated that MA2-24 possesses significant nootropic potential in domains of attention, cognitive flexibility, executive function and processing speed, as evidenced by increased correct responses, faster reaction times, and fewer errors when consumed both as a standalone intervention and in combination with caffeine.

**Clinical trial registration:**

https://ctri.nic.in/Clinicaltrials/pmaindet2.php?EncHid=MTE0NzIx&Enc=&userName=, identifier CTRI/2024/09/074472.

## 1 Introduction

In today’s fast-paced world with constant digital connectivity, information overload, high productivity demands, and psychological stress, maintaining optimal cognitive health has become increasingly important. However, cognitive performance may decline due to aging, adverse lifestyle factors, sleep disturbances, malnutrition, traumatic injury, metabolic disorders, neurocognitive diseases, chronic stress, and alcohol/drug use (American Psychiatric Association, 2013; World Health Organization, 2019; Livingston et al., 2020). The Diagnostic and Statistical Manual of Mental Disorders classifies cognition into six domains: learning and memory, complex attention, executive function, language, perceptual-motor control, and social cognition (American Psychiatric Association, 2013). Among these, two main components of growing public interest include attention/focus and reflex times for academic and professional advantage (Onaolapo et al., 2019). Attention refers to the ability to selectively focus on a specific stimulus while ignoring others, whereas reflex time is the interval between perceiving a stimulus and initiating a response (Shipp, 2004; McDowd, 2007). Both are essential for performance and executive functioning and are closely linked to alertness and mental energy.

Cognitive training and exercise, including yoga and meditation, along with supplementation of nootropics, have recently gained popularity for improving and/or maintaining cognitive performance among students, professionals, and athletes (Livingston et al., 2020). Nootropics are compounds such as vitamins, minerals, phytochemicals, and botanical extracts that may support alertness, attention, learning, and memory, while generally lacking the significant adverse effects associated with traditional stimulants (Malík and Tlustoš, 2022). Plant-based nootropics are generally considered safe and can improve cognition through multiple physiological mechanisms, including enhanced erythrocyte plasticity, increased cerebral blood flow, improved brain oxygen supply, and protection against oxidative stress–induced neuronal damage via antioxidant and anti-inflammatory effects (Malík and Tlustoš, 2023). They may also support the synthesis of neuronal proteins, nucleic acids, and phospholipids required for neurohormonal membrane repair. A recent review demonstrated that dietary alkaloids possess significant neuroprotective and cognition-enhancing properties by modulating glucose metabolism, oxidative stress, neuroinflammation, gut microbiota, and vascular function, supporting their potential as natural nootropic agents (Alkanad et al., 2024). In 2023, the global nootropics market was valued at approximately USD 14.25 billion and is projected to reach USD 51.83 billion by 2032, with a CAGR of about 15.4% (Zion Market Research, 2023).

Caffeine is the most widely used nootropic substance affecting the neurocognitive domains of attention and executive function. It enhances visual selective attention and improves behavioral performance accuracy and reaction speed (Rao et al., 2005; Kahathuduwa et al., 2018). In the central nervous system, caffeine stimulates dopaminergic and glutamatergic activity by antagonizing inhibitory adenosine A1 and A2 receptors (Ferré, 2008). Though oral doses of 200–400 mg can produce cognitive benefits typically lasting for 60–120 min, it is often followed by a “crash” phase characterized by fatigue (brain fog), irritability, headaches, insomnia, palpitations, gastrointestinal discomfort, and reduced concentration (Malinauskas et al., 2007; Herrick et al., 2009). Such dosage may also produce rapid inotropic and chronotropic cardiovascular effects such as increased heart rate and conductivity, along with heightened locomotor activity and anxiety-like symptoms (Cappelletti et al., 2015). These limitations have increased interest in caffeine-free plant nootropics that sustain focus and reduce fatigue (Lorca et al., 2023). Botanical extracts may act independently or synergistically with caffeine to increase synaptic acetylcholine, modulate monoamine oxidases, regulate glutamate receptors and reduce adenosine levels (Patel et al., 2024), thereby supporting low dose, extended duration use with minimal side effects.

Cardamom is a culinary spice and food additive by major food safety regulatory authorities worldwide, including the U.S. Food and Drug Administration, the European Food Safety Authority, Food Standards Australia and New Zealand, the Food Safety and Standards Authority of India, and the National Health Commission of the People’s Republic of China, along with other regulatory bodies across Asia. The two main species used worldwide are black cardamom (*Amomum subulatum* Roxb.) and green cardamom (*Elettaria cardamomum*). Both contain bioactive phytochemicals, including saponins, polyphenols, flavonoids, and essential oils rich in terpenes and terpenoids, which exhibit antioxidant, anti-inflammatory, and neuroprotective properties (Kabir et al., 2023; Dinata et al., 2024). Findings from our previously published acute clinical study demonstrated that the full-spectrum black cardamom extract (MA2-24) significantly enhanced attention, processing speed, cognitive flexibility, and executive function. Moreover, co-supplementation with caffeine potentiated these cognitive benefits while improving resilience to mental fatigue (Thomas et al., 2026). Pharmacological studies have further demonstrated that *A. subulatum* is of particular interest to brain functions. It enhances synaptic plasticity *via* upregulation of brain-derived neurotrophic factor (BDNF), improves cerebral blood flow, and modulates dopaminergic and cholinergic neurotransmission (Juergens, 2014; Kandikattu et al., 2017; Auti and Kulkarni, 2019; Saini et al., 2021; Abdel-Rasoul et al., 2023; Alruhaili et al., 2023; Ballester et al., 2023; Gazmer et al., 2023; Hoch et al., 2023; Bala et al., 2024). These mechanisms are central to attention, working memory, processing speed, and executive function. Cardamom has also been traditionally used as a nootropic in India and China (Kumar Singhal et al., 2022).

Based on its pharmacological profile and acute study evidence, it was hypothesized that the full-spectrum aqueous extract of black cardamom (MA2-24) would exert clinically relevant nootropic effects in healthy adults. Accordingly, a randomized, double-blind, placebo- and active-controlled clinical study was conducted to evaluate the effects of 14-day supplementation with MA2-24, caffeine, and their combination on cognitive performance across multiple domains, including attention, working memory, and processing speed. Cognitive performance was assessed using the computerized neurocognitive test battery Central Nervous System Vital Signs (CNSVS).

## 2 Materials and methods

### 2.1 Study material

Dried fruits of black cardamom were sourced from selected farms in Northeast India and taxonomically authenticated by a qualified botanist through comprehensive macroscopic and microscopic examination, DNA barcoding, and high-performance thin-layer chromatography (HPTLC; CAMAG, Muttenz, Switzerland) as described previously (Thomas et al., 2026). A voucher specimen (AKB-AS-03/24) was deposited at the Herbarium of the Akay Bioactives Innovation Centre, Kochi, India. The investigational materials, the standardized aqueous extract MA2-24 (Batch No. ASEU-010-SC-01/24; dated 08/09/2024) and natural caffeine derived from *Coffea arabica* beans (Batch No. CCAE-950-HC-02/24; dated 18/04/2024) were supplied by Akay Natural Ingredients, Kochi, India. Each material was accompanied by a certificate of analysis and a food safety data sheet confirming compliance with established quality standards and suitability for human consumption.

The investigational products (IP) were prepared as visually identical 3g sachets using predefined formulations as already reported (Thomas et al., 2026).

### 2.2 Ethical considerations

The study was conducted in accordance with Good Clinical Practice (GCP) guidelines and the ethical principles outlined in the Declaration of Helsinki, and complied with the Government of India’s clinical research regulations. Ethical approval was obtained from the registered Institutional Ethics Committee of BGS Global Institute of Medical sciences, Bangalore, Karnataka, India, and the protocol was registered with the Clinical Trial Registry of India (CTRI/2024/09/074472, dated 27/09/2024).

### 2.3 Sample size calculation

At the time of study planning, no published human clinical data were available on the cognitive effects of the proprietary black cardamom extract (MA2-24), making it impossible to estimate the expected effect size from previous studies. Therefore, the sample size calculation was based on Cohen’s conventional medium effect size (f = 0.25), which is widely recommended for superiority trials when reliable prior estimates are unavailable. This conservative assumption provides adequate statistical power while avoiding overestimation of treatment effects (Cohen, 1988; Faul et al., 2007; Weaver and Hodges, 2021). Assuming a medium effect size (Cohen’s f = 0.25), alpha = 0.05, power = 80%, four treatment groups, and five time points, a minimum total sample size of 84 participants was required. Allowing for approximately 10–15% attrition, 96 participants (24 per group) were enrolled to ensure adequate statistical power to detect clinically meaningful differences in cognitive performance.

### 2.4 Study design and protocol

A randomized, double-blind, placebo- and active-controlled, four-arm, parallel-group, 2-week clinical study was conducted to evaluate the effects of MA2-24, caffeine, and their combination on cognitive performance and physiological parameters in working professionals. Working professionals with elevated Fatigue Severity Scale (>4) were selected as they represent a relevant population for evaluating interventions intended to support sustained attention, processing speed, and executive performance. A total of 96 participants (59 males and 37 females), aged 35–65 years and meeting predefined eligibility criteria (Supplementary Table 1), were enrolled. Written informed consent was obtained from all participants prior to study participation.

The intervention period was set at 14 days based on evidence that cognitive benefits associated with botanical and nutraceutical interventions may be observed within 1–4 weeks of supplementation, whereas caffeine primarily exerts acute cognitive effects and may demonstrate reduced responsiveness with repeated consumption due to tolerance development (Fredholm et al., 1999; Juliano and Griffiths, 2004; Kennedy, 2014). Therefore, a 14-day supplementation period was considered an appropriate duration to evaluate the early efficacy, safety, and tolerability of MA2-24, while extending beyond single-dose assessments and providing preliminary evidence to support future longer-term investigations (Weaver and Hodges, 2021).

At the screening visit (Visit 1), demographic characteristics, medical history, physical examination findings, anthropometric measurements, and vital signs, including systolic and diastolic blood pressure, heart rate, and body temperature, were recorded (Supplementary Tables 2 and 3). The participants were dispensed with the test product as per randomization and were instructed to consume the IP for 14 days, 30 min after breakfast. They were instructed to abstain from caffeine and other stimulant-containing products for at least 48 h prior to intervention. During the visit days, the participants were provided with standardized breakfast at the site, post 30-min. To minimize the potential confounding effects of dietary composition, postprandial glycemic fluctuations, and nutrient-induced alterations in cognitive performance, all participants consumed a standardized breakfast before each cognitive assessment. The standardized meal consisted of two slices of whole wheat bread, one boiled egg, and 250 mL of water, providing approximately 250–300 kcal. The composition of the meal and timing were kept identical across all study visits and treatment groups to ensure consistency in metabolic status and to reduce inter- and intra-individual variability in cognitive outcomes. Participants remained at the study site for approximately 12 h to complete all scheduled assessments. During intervals between cognitive testing sessions, they were permitted to engage in quiet, non-strenuous activities, such as reading books or magazines, to minimize boredom while avoiding activities that could influence cognitive performance. Eligible participants proceeded to Visit 2, which involved a supervised 12-h in-clinic assessment.

The randomization sequence was generated by an independent statistician who was not involved in participant recruitment, clinical assessments, data collection, or data analysis. Predefined block sizes were concealed from the investigators to maintain allocation concealment until completion of the study. Participants were assigned sequentially according to the randomization schedule, and treatment allocation codes were securely maintained until completion of data analysis. Study products were identical in appearance, packaging, and labeling, ensuring blinding of participants, investigators, outcome assessors, and data analysts throughout the study. Participants were randomized in an equal allocation ratio (n = 24 per group) using a permuted-block randomization scheme to receive placebo (250 mg), MA2-24 (250 mg), caffeine (200 mg), or MA2-24 combined with caffeine (250 mg + 200 mg).

Baseline assessments were conducted under fasting conditions. The investigational products were administered once daily, as identical sachets dissolved in 200 ± 20 mL of water. Participant compliance was assessed at the end of the study by counting the unused sachets returned by each participant. Compliance was calculated based on the number of sachets consumed relative to the number dispensed. Vital signs and cognitive performance, assessed using the CNS Vital Signs (CNSVS) computerized neurocognitive test battery, were evaluated at baseline and at 1, 3, 5, and 8 h post-dose on days 1, 7, and 14. Adverse events were systematically recorded throughout the study period. Participant disposition and study design are presented in Figure 1A and timeline of study visit procedures and cognitive assessments are illustrated in Figure 1B.

**FIGURE 1:**
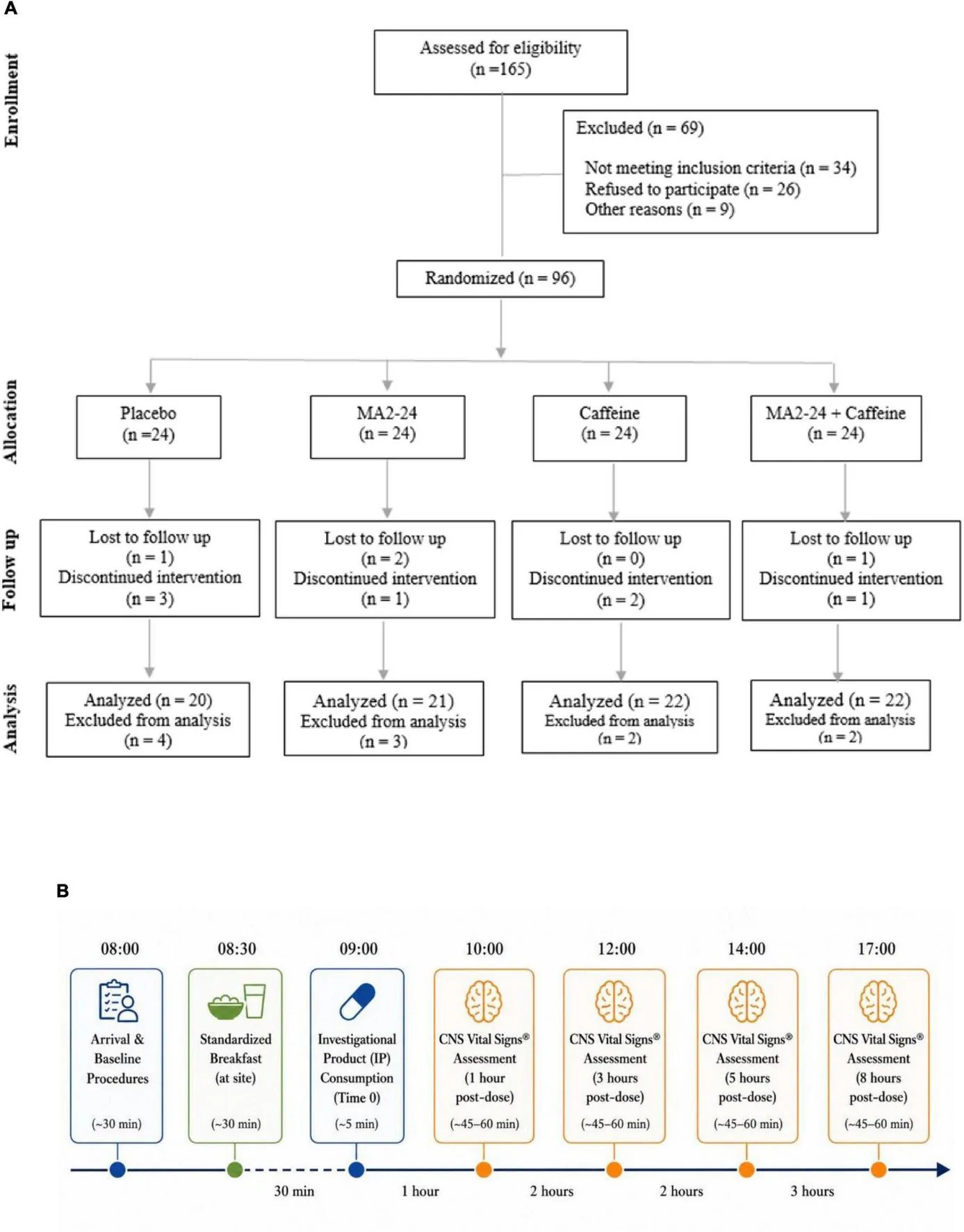
**(A)** CONSORT flow diagram depicting participant enrollment, allocation, follow-up, and analysis, along with a schematic overview of the cohort design, **(B)** Timeline of study visit procedures and cognitive assessments.

#### 2.4.1 CNSVS

CNSVS assessments are extensively validated, robust computerized neuropsychological tests that measure the neurocognitive status of a subject (Gualtieri and Johnson, 2006). The primary outcome measure of the study was to evaluate the effect of MA2-24 as compared to placebo on cognitive performance using validated CNSVS tests, including the Shifting Attention Test (SAT), Symbol Digit Coding (SDC), and Stroop Test (ST), to assess simple and complex reaction time, processing speed, and executive function. The tests were conducted as explained previously (Thomas et al., 2026). Participants received instructions on CNS Vital Signs (CNSVS) assessments during the screening visit (Visit 1) before any baseline evaluations were conducted. In addition, the CNSVS battery incorporates built-in practice trials for each cognitive test, allowing participants to become familiar with the test procedures before the actual assessment. Furthermore, the randomized, double-blind, placebo-controlled study design ensured that any potential practice or learning effects associated with repeated cognitive testing were equally distributed across all treatment groups.

### 2.5 Safety and tolerance

The secondary outcomes of safety and tolerability were evaluated throughout the study period by monitoring adverse events, physical examinations, and vital sign measurements.

### 2.6 Statistical analysis

Statistical analyses were performed using the R software (version 4.3.3). Data processing and visualization were conducted using the tidyverse package, while treatment comparisons were carried out using the emmeans, multcomp, and multcompView packages. Treatment effects were evaluated using a three-way factorial linear model with treatment group, study day, and assessment time as fixed factors, along with their interaction terms (Group × Day × Time). Estimated marginal means (EMMs) were calculated to obtain adjusted group means across time points. *Post hoc* comparisons between each treatment group and placebo were performed using Dunnett’s multiple comparison test, which controls the family-wise error rate when comparing multiple treatments to a single control. Statistical significance was set at *p* < 0.05. For graphical representation, boxplots with jittered individual observations and mean values were generated using ggplot2. Significance groupings were annotated using compact letter displays derived from Dunnett-adjusted comparisons.

## 3 Results

A total of 165 participants were screened for eligibility, of whom 69 were excluded due to not meeting the inclusion criteria. A total of 96 eligible participants were randomized equally into four intervention groups. During the 14-day intervention period, eleven participants discontinued the study due to loss to follow-up or discontinuation of intervention. The final analysis included 85 participants, comprising 20 participants in the placebo group, 21 in the MA2-24 group, 22 in the caffeine group, and 22 in the MA2-24 plus caffeine group. The participant flow and disposition throughout the study are presented in the CONSORT flow diagram (Figure 1A)

Based on sachet count at the end of the study (Day 14), overall compliance was 99.8% in the MA2-24 group, 100% in the MA2-24 plus caffeine group, 99.4% in the caffeine group, and 100% in the placebo group, indicating excellent adherence to the assigned interventions across all treatment groups.

### 3.1 Primary outcomes

#### 3.1.1 SAT

##### 3.1.1.1 SAT-correct responses

A linear mixed-effects model revealed significant main effects of treatment group [F(3, 94.85) = 37.86, *p* < *0.001*, partial η^2^ = 0.545], study day [F(2, 990.13) = 68.45, *p* < *0.001*, partial η^2^ = 0.121], and post-dose assessment time [F(3, 962.47) = 88.25, *p* < *0.001*, partial η^2^ = 0.216], indicating that SAT correct responses differed across treatment groups, study days, and assessment time points. Significant Group × Day [F(6, 989.75) = 13.64, *p* < *0.001*, partial η^2^ = 0.076], Group × Time [F(9, 962.47) = 6.56, *p* < *0.001*, partial η^2^ = 0.058], and Day × Time [F(6, 962.47) = 21.92, *p* < *0.001*, partial η^2^ = 0.120] interactions were also observed, whereas the three-way interaction (Group × Day × Time) was not statistically significant [F(18, 962.47) = 1.38, *p* = 0.135, partial η^2^ = 0.025].

Subsequent Dunnett-adjusted *post hoc* comparisons demonstrated that improvements in SAT correct responses were most pronounced during the early post-dose assessments (Figure 2A; Supplementary Table 4). During Day 1 assessment, all active treatments significantly improved SAT correct responses at both 1 h and 3 h compared with placebo. At 5 h, the beneficial effect was retained only with the combined MA2-24 + caffeine treatment (estimate = 4.50, *p* = 0.025), indicating an earlier decline in the effects of the individual interventions.

**FIGURE 2:**
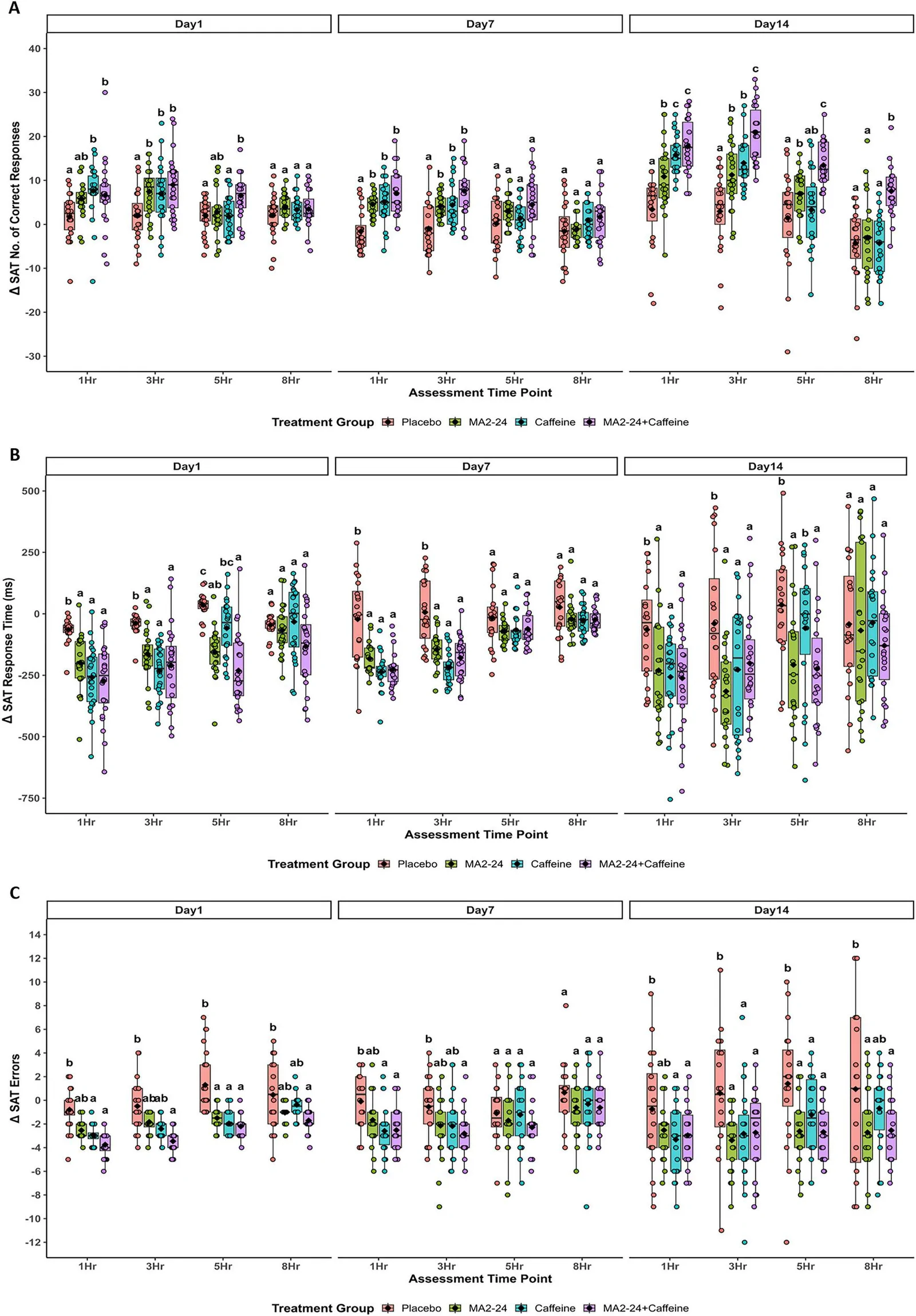
Effects of treatments on SAT performance: **(A)** SAT correct responses, **(B)** SAT reaction time, and **(C)** SAT errors. Assessments were conducted on day 1, day 7, and day 14 at 1 h, 3 h, 5 h, and 8 h post-dose. Boxplots represent data distribution with individual observations and mean values. Statistical comparisons were performed using a repeated-measures mixed-effects model followed by Dunnett’s *post hoc*-test against the placebo group. Lowercase letters (a, b, c) above the boxplots indicate significant differences between groups within the same day and time point (*p* < 0.05). Groups sharing at least one common letter are not significantly different, whereas groups with different letters are significantly different.

By Day 7, improvements at 1 h were maintained with MA2-24 (estimate = 6.17, *p* = 0.003), caffeine (estimate = 6.65, *p* < 0.001), and were greatest with the combined treatment (estimate = 8.76, *p* < 0.001). All three interventions also remained superior to placebo at 3 h. By 5 h, only the combined treatment continued to demonstrate a significant benefit (estimate = 4.39, *p* = 0.049), suggesting a more sustained effect of co-supplementation.

By Day 14, the strongest treatment effects were observed on the final study day. At 1 h, SAT correct responses increased significantly with MA2-24 (estimate = 7.46, *p* < 0.001), caffeine (estimate = 12.36, *p* < 0.001), and particularly with the combined treatment (estimate = 14.28, *p* < 0.001). Similar improvements were evident at 3 h, where the combination again produced the largest effect (estimate = 17.91, *p* < 0.001), exceeding the improvements observed with MA2-24 (estimate = 8.29, *p* < 0.001) and caffeine (estimate = 10.99, *p* < 0.001). At 5 h, significant improvements persisted with MA2-24 (estimate = 5.55, *p* = 0.008), while the combined treatment showed a substantially greater benefit (estimate = 11.96, *p* < 0.001). Notably, at 8 h, the combined MA2-24 + caffeine treatment remained the only intervention demonstrating a significant improvement over placebo (estimate = 11.80, *p* < 0.001), indicating the most sustained enhancement in sustained attention.

##### 3.1.1.2 SAT-reaction time

Analysis using a linear mixed-effects model showed significant main effects of treatment group [F(3, 88.51) = 22.61, *p* < *0.001*, partial η^2^ = 0.434], study day [F(2, 990.58) = 13.28, *p* < *0.001*, partial η^2^ = 0.026], and post-dose assessment time [F(3, 963.83) = 52.18, *p* < *0.001*, partial η^2^ = 0.140]. Significant Group × Day [F(6, 990.11) = 3.06, *p* = 0.006, partial η^2^ = 0.018] and Group × Time [F(9, 963.83) = 6.39, *p* < *0.001*, partial η^2^ = 0.056] interactions indicated that treatment effects differed across study days and post-dose assessment times. No significant Day × Time [F(6, 963.83) = 0.42, *p* = 0.868, partial η^2^ = 0.003] or Group × Day × Time [F(18, 963.83) = 1.20, *p* = 0.253, partial η^2^ = 0.022] interactions were recorded.

Dunnett-adjusted *post hoc* comparisons demonstrated significantly lower SAT reaction times in the active treatment groups than in the placebo group across multiple study days and post-dose assessment time points (Figure 2B; Supplementary Table 5). On Day 1, all active treatments significantly reduced SAT reaction time at both 1 h (MA2-24: estimate = −134.21, *p* = 0.010; caffeine: estimate = −193.63, *p* < 0.001; MA2-24 + caffeine: estimate = −208.71, *p* < 0.001) and 3 h (MA2-24: estimate = −124.92, *p* = 0.019; caffeine: estimate = −194.38, *p* < 0.001; MA2-24 + caffeine: estimate = −172.17, *p* < 0.001) compared with placebo. At 5 h, significant reductions were observed with MA2-24 (estimate = −191.67, *p* < 0.001) and the combined treatment (estimate = −266.29, *p* < 0.001).

On Day 7, all active treatments remained significantly lower than placebo at both 1 h (MA2-24: estimate = −161.16, *p* = 0.004; caffeine: estimate = −212.73, *p* < 0.001; MA2-24 + caffeine: estimate = −195.92, *p* < 0.001) and 3 h (MA2-24: estimate = −151.87, *p* = 0.007; caffeine: estimate = −222.64, *p* < 0.001; MA2-24 + caffeine: estimate = −176.97, *p* < 0.001). No significant differences were observed at 5 h or 8 h.

During Day 14, all active treatments significantly reduced SAT reaction time at both 1 h (MA2-24: estimate = −165.69, *p* = 0.003; caffeine: estimate = −190.61, *p* < 0.001; MA2-24 + caffeine: estimate = −188.76, *p* < 0.001) and 3 h, with estimates of −275.68 (*p* < 0.001), −185.73 (*p* < 0.001), and −152.70 (*p* = 0.006) for MA2-24, caffeine, and MA2-24 + caffeine, respectively. At 5 h, significant reductions were observed with MA2-24 (estimate = −243.62, *p* < 0.001) and the combined treatment (estimate = −251.91, *p* < 0.001). No significant differences were observed at 8 h.

##### 3.1.1.3 SAT-errors

Mixed-effects analysis demonstrated that SAT Δ errors were significantly influenced by treatment group [F(3, 97.24) = 22.19, *p* < *0.001*, partial η^2^ = 0.406] and post-dose assessment time [F(3, 968.22) = 21.36, *p* < *0.001*, partial η^2^ = 0.062]. A significant Group × Day interaction was also observed [F(6, 993.05) = 3.37, *p* = 0.003, partial η^2^ = 0.020], whereas the main effect of study day and the Group × Time, Day × Time, and three-way interactions were not statistically significant.

During Day 1, caffeine (estimate = −2.17, *p* = 0.014) and the combined MA2-24 + caffeine treatment (estimate = −2.96, *p* < 0.001) significantly reduced Δ errors at both 1 h and 3 h compared with placebo. By 5 h, significant reductions were observed in all three treatment groups, including MA2-24 (estimate = −2.79, *p* < 0.001), caffeine (estimate = −3.25, *p* < 0.001), and MA2-24 + caffeine (estimate = −3.50, *p* < 0.001). At the final assessment (8 h), only the combined treatment remained significantly different from placebo (estimate = −2.17, *p* = 0.014) (Figure 2C; Supplementary Table 6).

On Day 7, significant reductions at 1 h were observed with caffeine (estimate = −2.60, *p* = 0.005) and the combined treatment (estimate = −2.59, *p* = 0.005), whereas at 3 h only the combined treatment remained significant (estimate = −2.42, *p* = 0.010). No significant treatment effects were detected at the later assessment times.

By Day 14, treatment effects were evident across multiple post-dose assessments. At 1 h, caffeine (estimate = −2.67, *p* = 0.003) and the combined treatment (estimate = −2.35, *p* = 0.012) significantly reduced Δ errors. At both 3 h and 5 h, all active treatments demonstrated significant reductions relative to placebo, with MA2-24 (3 h: estimate = −4.12, *p* < 0.001; 5 h: estimate = −4.16, *p* < 0.001) and the combined treatment (3 h: estimate = −3.47, *p* < 0.001; 5 h: estimate = −4.23, *p* < 0.001) showing the largest reductions. At 8 h, significant reductions persisted for MA2-24 (estimate = −3.80, *p* < 0.001) and the combined treatment (estimate = −3.64, *p* < 0.001).

#### 3.1.2 SDC

##### 3.1.2.1 SDC-correct responses

Evaluation of SDC correct responses using a linear mixed-effects model revealed significant effects of treatment group [F(3, 92.23) = 61.04, *p* < *0.001*, partial η^2^ = 0.665], study day [F(2, 863.62) = 7.42, *p* < *0.001*, partial η^2^ = 0.017], and post-dose assessment time [F(3, 938.07) = 102.19, *p* < *0.001*, partial η^2^ = 0.246]. Significant Group × Day [F(6, 774.19) = 5.78, *p* < *0.001*, partial η^2^ = 0.043], Group × Time [F(9, 938.07) = 11.31, *p* < *0.001*, partial η^2^ = 0.098], Day × Time [F(6, 938.07) = 5.14, *p* < *0.001*, partial η^2^ = 0.032], and Group × Day × Time interactions [F(18, 938.07) = 1.73, *p* = 0.030, partial η^2^ = 0.032] were also observed.

Across the first study day, all active treatments significantly improved SDC correct responses at both 1 h (MA2-24: estimate = 5.71, *p* < 0.001; caffeine: estimate = 7.54, *p* < 0.001; MA2-24 + caffeine: estimate = 11.45, *p* < 0.001) and 3 h (MA2-24: estimate = 7.79, *p* < 0.001; caffeine: estimate = 10.54, *p* < 0.001; MA2-24 + caffeine: estimate = 13.07, *p* < 0.001). At 5 h, significant improvements were maintained with MA2-24 (estimate = 4.25, *p* = 0.017) and the combined treatment (estimate = 14.24, *p* < 0.001) (Figure 3A; Supplementary Table 7).

**FIGURE 3:**
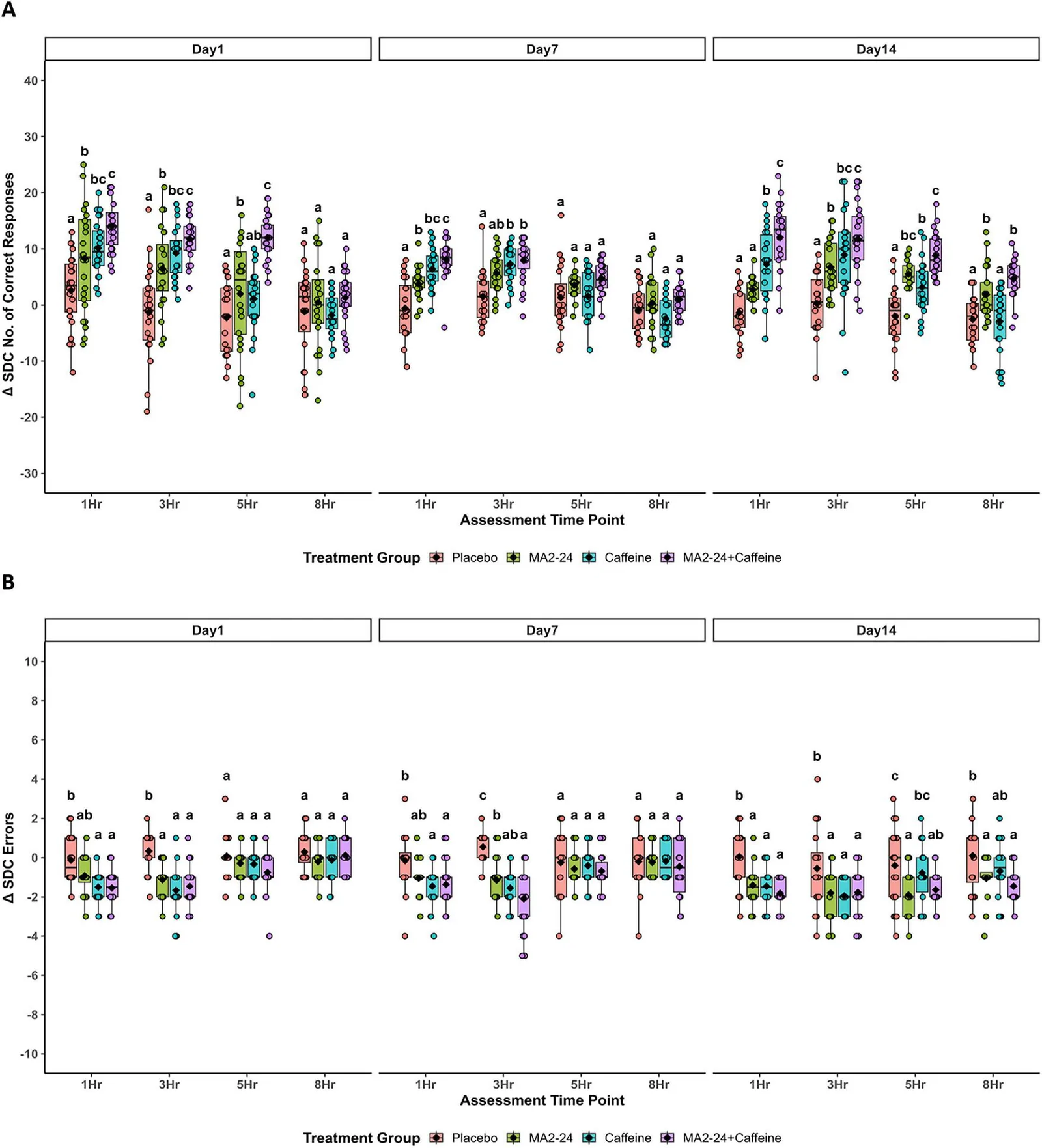
Effects of treatments on SDC performance: **(A)** SDC correct responses and **(B)** SDC errors, measured on day 1, day 7, and day 14 at 1 h, 3 h, 5 h, and 8 h post-dose. Boxplots represent data distribution with individual observations and mean values. Statistical comparisons were performed using a repeated-measures mixed-effects model followed by Dunnett’s *post hoc*-test against the placebo group. Lowercase letters (a, b, c) above the boxplots indicate significant differences between groups within the same day and time point (*p* < 0.05). Groups sharing at least one common letter are not significantly different, whereas groups with different letters are significantly different.

A comparable pattern was observed on Day 7, with all three treatments remaining significantly superior to placebo at both 1 h (MA2-24: estimate = 4.29, *p* = 0.028; caffeine: estimate = 6.87, *p* < 0.001; MA2-24 + caffeine: estimate = 8.55, *p* < 0.001) and 3 h (MA2-24: estimate = 4.05, *p* = 0.042; caffeine: estimate = 5.63, *p* = 0.002; MA2-24 + caffeine: estimate = 6.22, *p* < 0.001). No significant differences were detected at the later assessments.

By the final study day (Day 14), significant improvements were evident at all earlier post-dose assessments. At 1 h, correct responses increased with MA2-24 (estimate = 4.04, *p* = 0.043), caffeine (estimate = 8.48, *p* < 0.001), and MA2-24 + caffeine (estimate = 13.25, *p* < 0.001). Similar findings were observed at 3 h (MA2-24: estimate = 6.44, *p* < 0.001; caffeine: estimate = 8.57, *p* < 0.001; MA2-24 + caffeine: estimate = 11.38, *p* < 0.001) and 5 h (MA2-24: estimate = 7.40, *p* < 0.001; caffeine: estimate = 4.99, *p* = 0.007; MA2-24 + caffeine: estimate = 10.63, *p* < 0.001). At 8 h, significant improvements persisted with MA2-24 (estimate = 4.34, *p* = 0.026) and the combined treatment (estimate = 7.09, *p* < 0.001).

##### 3.1.2.2 SDC-errors

Analysis of SDC Δ errors using a linear mixed-effects model revealed significant main effects of treatment group [F(3, 94.88) = 24.63, *p* < *0.001*, partial η^2^ = 0.438], study day [F(2, 991.74) = 30.39, *p* < *0.001*, partial η^2^ = 0.058], and post-dose assessment time [F(3, 965.87) = 38.37, *p* < *0.001*, partial η^2^ = 0.106]. Group × Time [F(9, 965.87) = 6.80, *p* < *0.001*, partial η^2^ = 0.060], Day × Time [F(6, 965.87) = 2.51, *p* = 0.021, partial η^2^ = 0.015], and Group × Day × Time [F(18, 965.87) = 1.72, *p* = 0.031, partial η^2^ = 0.031] interactions were significant, whereas the Group × Day interaction [F(6, 991.26) = 2.01, *p* = 0.062, partial η^2^ = 0.012] was not significant.

Treatment-related differences in SDC errors relative to placebo across several post-dose assessment time points and study days were observed (Figure 3B, Supplementary Table 8). During the first study day, all active treatments significantly reduced SDC Δ errors at both 1 h (MA2-24: estimate = −0.79, *p* = 0.041; caffeine: estimate = −1.38, *p* < 0.001; MA2-24 + caffeine: estimate = −1.42, *p* < 0.001) and 3 h (MA2-24: estimate = −1.46, *p* < 0.001; caffeine: estimate = −2.00, *p* < 0.001; MA2-24 + caffeine: estimate = −1.79, *p* < 0.001). At 5 h, a significant reduction was observed only with the combined treatment (estimate = −0.83, *p* = 0.029).

The corresponding assessments on Day 7 showed significant reductions at both 1 h (MA2-24: estimate = −0.89, *p* = 0.031; caffeine: estimate = −1.33, *p* < 0.001; MA2-24 + caffeine: estimate = −1.22, *p* < 0.001) and 3 h (MA2-24: estimate = −1.68, *p* < 0.001; caffeine: estimate = −2.12, *p* < 0.001; MA2-24 + caffeine: estimate = −2.64, *p* < 0.001) for all active treatments. No significant differences were observed at 5 h or 8 h.

During the final study day (Day 14), all active treatments significantly reduced SDC Δ errors at both 1 h (MA2-24: estimate = −1.41, *p* < 0.001; caffeine: estimate = −1.53, *p* < 0.001; MA2-24 + caffeine: estimate = −1.87, *p* < 0.001) and 3 h (MA2-24: estimate = −1.21, *p* = 0.002; caffeine: estimate = −1.43, *p* < 0.001; MA2-24 + caffeine: estimate = −1.23, *p* < 0.001). At the later assessments, significant reductions were observed with MA2-24 and the combined treatment at 5 h (MA2-24: estimate = −1.46, *p* < 0.001; MA2-24 + caffeine: estimate = −1.24, *p* < 0.001) and 8 h (MA2-24: estimate = −1.11, *p* = 0.005; MA2-24 + caffeine: estimate = −1.56, *p* < 0.001).

#### 3.1.3 ST

##### 3.1.3.1 Stroop simple-reaction time

A linear mixed-effects model revealed significant main effects of Group [F(3, 91.03) = 22.96, *P* < 0.001, partial η^2^ = 0.431], Day [F(2, 991.75) = 20.58, *P* < 0.001, partial η^2^ = 0.040], and Time [F(3, 966.07) = 229.47, *P* < 0.001, partial η^2^ = 0.416]. Significant Group × Day [F(6, 991.30) = 6.41, *P* < 0.001, partial η^2^ = 0.037], Group × Time [F(9, 966.07) = 10.65, *P* < 0.001, partial η^2^ = 0.090], Day × Time [F(6, 966.07) = 4.27, *P* < 0.001, partial η^2^ = 0.026], and Group × Day × Time [F(18, 966.07) = 2.31, *P* = 0.002, partial η^2^ = 0.041] interactions were observed. Analysis of Stroop simple reaction time demonstrated treatment-associated changes relative to placebo across several study days and post-dose time points (Figure 4A; Supplementary Table 9). On Day 1, significant reductions in reaction time were observed at 1 h with MA2-24 (estimate = −53.54, *P* = 0.036), caffeine (estimate = −62.00, *P* = 0.011), and MA2-24 + caffeine (estimate = −112.29, *P* < 0.001). At 3 h, significant reductions were observed with caffeine (estimate = −54.79, *P* = 0.030) and MA2-24 + caffeine (estimate = −102.54, *P* < 0.001). At 5 h, a significant reduction was observed only with MA2-24 + caffeine (estimate = −67.21, *P* = 0.005). No significant differences were observed at 8 h.

**FIGURE 4:**
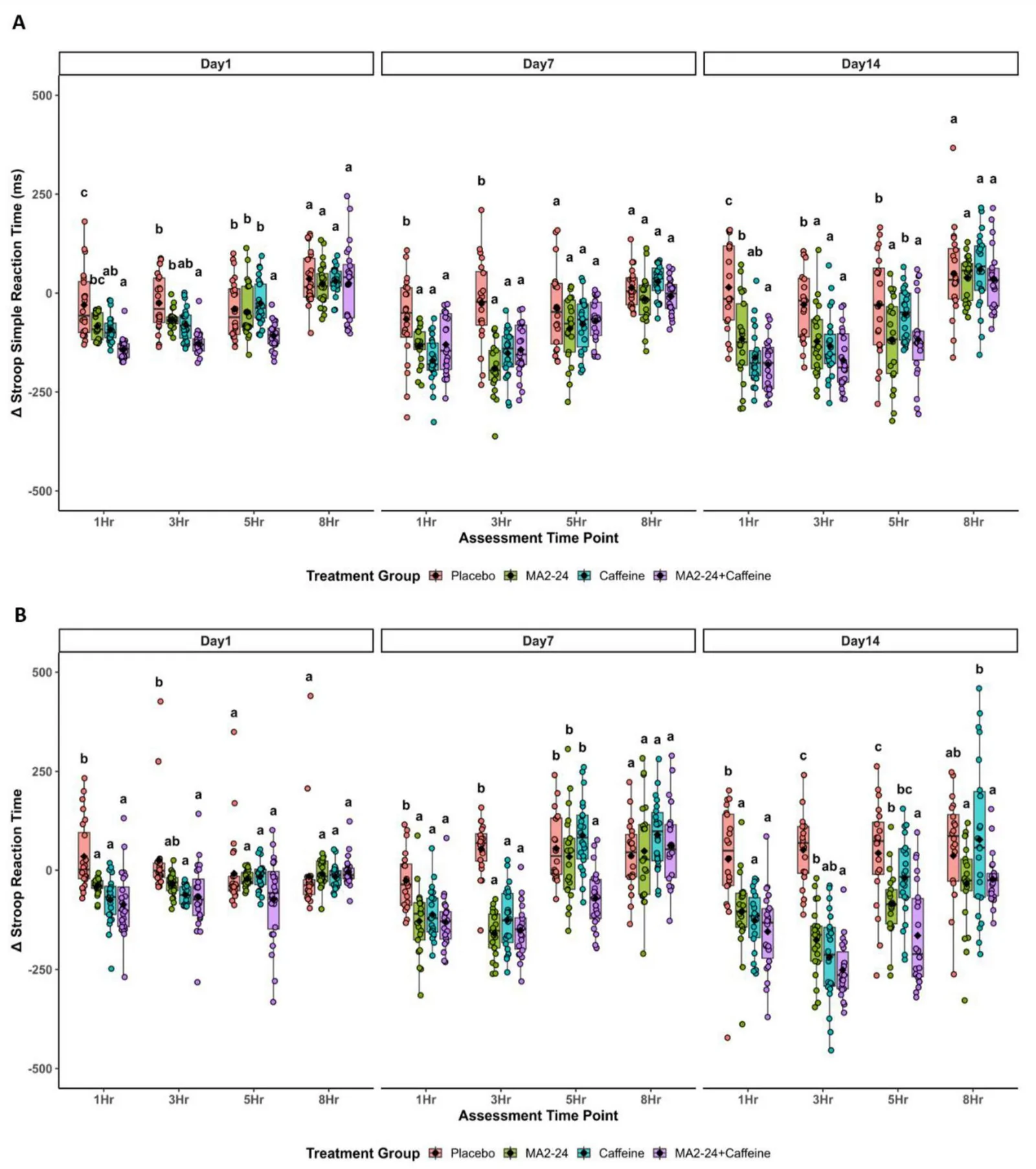
Effects of treatments on Stroop performance: **(A)** Stroop simple reaction time and **(B)** Stroop reaction time, assessed on day 1, day 7, and day 14 at 1 h, 3 h, 5 h, and 8 h post-dose. Boxplots represent data distribution with individual observations and mean values. Statistical comparisons were performed using a repeated-measures mixed-effects model followed by Dunnett’s *post hoc*-test against the placebo group. Lowercase letters (a, b, c) above the boxplots indicate significant differences between groups within the same day and time point (*p* < 0.05). Groups sharing at least one common letter are not significantly different, whereas groups with different letters are significantly different.

On Day 7, significant reductions in reaction time were observed at 1 h with MA2-24 (estimate = −65.78, *P* = 0.013), caffeine (estimate = −102.93, *P* < 0.001), and MA2-24 + caffeine (estimate = −61.30, *P* = 0.021). At 3 h, all three active treatments showed significantly lower reaction times than placebo, including MA2-24 (estimate = −165.15, *P* < 0.001), caffeine (estimate = −126.56, *P* < 0.001), and MA2-24 + caffeine (estimate = −116.34, *P* < 0.001). No significant differences were observed at 5 h or 8 h.

On Day 14, significant reductions in reaction time were observed at 1 h with MA2-24 (estimate = −129.62, *P* < 0.001), caffeine (estimate = −175.90, *P* < 0.001), and MA2-24 + caffeine (estimate = −192.81, *P* < 0.001). At 3 h, significant reductions were observed with MA2-24 (estimate = −91.72, *P* < 0.001), caffeine (estimate = −102.88, *P* < 0.001), and MA2-24 + caffeine (estimate = −137.52, *P* < 0.001). At 5 h, significant reductions were observed with MA2-24 (estimate = −88.15, *P* < 0.001) and MA2-24 + caffeine (estimate = −84.29, *P* < 0.001). No significant differences were observed at 8 h.

##### 3.1.3.2 Stroop-reaction time

Analysis of Stroop reaction time using a linear mixed-effects model revealed significant main effects of treatment group [F(3, 97.26) = 33.74, *p* < *0.001*, partial η^2^ = 0.510], study day [F(2, 997.10) = 28.73, *p* < *0.001*, partial η^2^ = 0.054], and post-dose assessment time [F(3, 972.68) = 103.79, *p* < *0.001*, partial η^2^ = 0.242]. Significant Group × Day [F(6, 996.67) = 9.93, *p* < *0.001*, partial η^2^ = 0.056], Group × Time [F(9, 972.68) = 19.11, *p* < *0.001*, partial η^2^ = 0.150], Day × Time [F(6, 972.68) = 21.17, *p* < *0.001*, partial η^2^ = 0.115], and Group × Day × Time [F(18, 972.68) = 2.26, *p* = 0.002, partial η^2^ = 0.040] interaction effects were statistically significant.

Following the first dose (Day 1), significant reductions were observed at 1 h for MA2-24 (estimate = −75.71, *p* = 0.013), caffeine (estimate = −109.38, *p* < 0.001), and MA2-24 + caffeine (estimate = −121.58, *p* < 0.001). At 3 h, significant differences were detected for caffeine (estimate = −86.96, *p* = 0.003) and MA2-24 + caffeine (estimate = −92.04, *p* = 0.002). By 5 h, only the combined treatment remained significantly different from placebo (estimate = −65.29, *p* = 0.039), whereas no significant differences were observed at 8 h. (Figure 4B, Supplementary Table 10).

After 1 week of intervention (Day 7), all three treatments were significantly different from placebo at both 1 h (MA2-24: estimate = −99.44, *p* = 0.002; caffeine: estimate = −82.46, *p* = 0.011; MA2-24 + caffeine: estimate = −102.84, *p* < 0.001) and 3 h (MA2-24: estimate = −210.98, *p* < 0.001; caffeine: estimate = −175.96, *p* < 0.001; MA2-24 + caffeine: estimate = −203.93, *p* < 0.001). At 5 h, significance was observed only for the combined treatment (estimate = −124.68, *p* < 0.001), while no significant differences were detected at 8 h.

At the end of the intervention (Day 14), all active treatments showed significant reductions in reaction time at 1 h (MA2-24: estimate = −130.25, *p* < 0.001; caffeine: estimate = −151.74, *p* < 0.001; MA2-24 + caffeine: estimate = −180.43, *p* < 0.001) and 3 h (MA2-24: estimate = −225.40, *p* < 0.001; caffeine: estimate = −267.35, *p* < 0.001; MA2-24 + caffeine: estimate = −300.91, *p* < 0.001). Significant reductions at 5 h were observed with MA2-24 (estimate = −126.39, *p* < 0.001) and MA2-24 + caffeine (estimate = −206.47, *p* < 0.001). At 8 h, a significant difference from placebo was observed only for MA2-24 (estimate = −68.59, *p* = 0.046).

### 3.4 Safety and tolerance

Safety and tolerability were systematically evaluated throughout via physical examinations and participant-reported outcomes. MA2-24 was well tolerated, with no clinically significant abnormalities observed during the post-intervention assessments. A total of 17 participants (17.7%) reported 22 adverse events (AEs) during the study. The incidence of AEs was highest in the caffeine group (12 participants; 50%), followed by the MA2-24 + caffeine group (4 participants; 16.7%), placebo (1 participant; 4.2%), while no AEs were reported in the MA2-24 group. The most frequently reported AEs were increased heart rate, gastrointestinal disturbance, sleep disturbance, and increased urination. All AEs were mild, resolved during the study, and no serious adverse events or treatment discontinuations were reported due to adverse events.

## 4 Discussion

The growing interest in natural nootropics reflects an increasing preference for holistic, sustainable, and safe approaches to cognitive health and overall well-being (Lorca et al., 2023; Chaachouay and Zidane, 2024). In this context, the present randomized, double-blind, placebo-controlled clinical study evaluated the acute and repeated-dose effects of the full-spectrum aqueous black cardamom extract, MA2-24, on cognitive performance over a 14-day intervention period, with caffeine and placebo included as comparator groups. Cognitive performance was assessed using the CNSVS test battery, including the SAT, SDC, and Stroop Test. Together, these tasks evaluated attention shifting, psychomotor and information processing speed, cognitive flexibility, and executive control (Thomas et al., 2026; Pillay et al., 2024).

Across these cognitive domains, MA2-24 produced significant improvements in performance, evidenced by faster reaction times, greater response accuracy, and reduced error rates, indicating enhanced attentional processing, cognitive flexibility, and executive functioning. In the early post-dose period, at hours 1 and 3, MA2-24 showed effects that were often comparable to caffeine across several outcomes. Co-administration with caffeine generally resulted in greater and/or more sustained benefits (5 to 8 h) in reaction time, correct responses, and errors. Collectively, these results support the cognitive-enhancing potential of MA2-24, alone or in conjunction with caffeine, across repeated dosing.

Improvements were observed across SAT, SDC, and Stroop outcomes, although the magnitude and persistence varied by endpoint and post-dose time point. SAT correct responses also improved at several time points, particularly at 1 and 3-h post-dose across study days, with additional benefits observed at 5-h post-dose on day 14. MA2-24 improved SAT reaction time across all study days, mainly at 1 and 3-h post-dose, with select benefits extending to 5-h. SAT error reductions were more selective, with clearer improvements emerging at specific time points, particularly by day 14. MA2-24 also increased SDC correct responses and reduced SDC errors at several time points, most consistently at 1 and 3-h post-dose, with some residual effects observed at 5-h and limited evidence at 8-h, primarily on day 14. MA2-24 also improved Stroop reaction times across multiple assessments, with stronger and more consistent effects generally observed on day 14.

The cognitive effects of MA2-24 may be partly attributed to the neuroprotective and neuromodulatory properties of cardamom-derived phytochemicals, including saponins, polyphenols, flavonoids, and terpenoids. Cardamom extracts exhibit antioxidant and anti-inflammatory activities that may protect neurons from oxidative stress and modulate neurotransmitter systems involved in cognition (Majdalawieh and Carr, 2010; Abdel-Rasoul et al., 2023). Preclinical studies in Wistar rats have demonstrated that supplementation with cardamom improved learning and memory performance in the T-maze and radial arm maze paradigms (Setty et al., 2013). Additionally, cardamom extracts reduce acetylcholinesterase activity in a dose-dependent manner, enhancing cholinergic signaling associated with cognitive function (Saini et al., 2021). Cardamom essential oil is rich in 1,8-cineole, a monoterpene (Satyal et al., 2012), which has demonstrated neuroprotective effects in animal models, including inhibition of acetylcholinesterase, reduction of oxidative damage, attenuation of neuronal degeneration, and suppression of amyloid-β deposition (Auti and Kulkarni, 2019). MA2-24 is a full-spectrum extract characterized by a high saponin content (>25% w/w), measurable levels of polyphenols and flavonoids, and ≥ 2.5% 1,8-cineole. Collectively, this profile suggests that, unlike caffeine’s primary action as an adenosine receptor antagonist, MA2-24 may modulate cognitive function through complementary mechanisms involving antioxidant activity, neurotransmitter regulation, and cholinergic modulation.

An additional observation was the prolonged cognitive effect in the co-supplementation group receiving MA2-24 + caffeine. This group showed improved performance across multiple time points, including higher accuracy, lower error rates, and faster stroop reaction times, particularly at 5 and 8-h post-administration. Although the magnitude of change across days 1, 7, and 14 did not differ significantly within the combination group, performance remained consistently superior to placebo and caffeine, indicating sustained effects. These findings suggest a potential synergistic interaction between MA2-24 and caffeine, enhancing and extending caffeine-induced cognitive benefits. Sustained improvements in accuracy, error reduction, and processing speed further support the potential of this combination to provide more durable cognitive enhancement or enable reduced caffeine dosing. Notably, some other multi-component nootropic formulations tested previously have not demonstrated benefits beyond caffeine in controlled studies (Caldenhove et al., 2018).

The prolonged cognitive benefits observed with co-supplementation may help mitigate the well-documented post-caffeine decline in performance. Caffeine enhances vigilance and alertness, partly via reductions in tonic α-band activity in the frontoparietal cortex, as shown in electroencephalographic studies (Dager and Friedman, 2000). It is rapidly absorbed, reaching peak plasma levels within 15–120 min (Fisone et al., 2004). As caffeine is metabolized, adenosine accumulation may contribute to a decline in alertness and mental energy, commonly termed the caffeine crash (Malinauskas et al., 2007; Herrick et al., 2009). In this study, sustained cognitive improvements with co-administration of MA2-24 and caffeine suggest that MA2-24 may prolong caffeine-induced alertness beyond its typical duration. This interaction may help extend the cognitive performance window when used with caffeine, potentially enabling a lower dose and reducing fatigue associated with caffeine metabolism. However, the underlying mechanisms require further investigation.

Consistent improvements in behavioral outcomes throughout the 14-day intervention period support the efficacy of MA2-24 in enhancing cognitive performance. These results suggest its potential utility as a functional cognitive ingredient. However, the relatively short duration limits the ability to draw conclusions about its long-term efficacy. Furthermore, the assessment was restricted to behavioral measures, without incorporating neurochemical or biomarker analyses, thereby limiting mechanistic interpretation. Future studies integrating neurophysiological assessments and including larger sample sizes are warranted to establish the underlying mechanisms and evaluate sustained cognitive benefits.

In addition to its efficacy, MA2-24 exhibited a favorable safety and tolerability profile, with no adverse events reported during the intervention period. This is consistent with existing evidence indicating that cardamom essential oil is well tolerated, with reported safety at doses up to 2000 mg/kg body weight in animal models (Manjunath and Mahurkar, 2021). Nevertheless, long-term safety evaluations in human populations remain necessary to confirm its safety profile with prolonged use.

## 5 Conclusion

The present study suggests that MA2-24, a full-spectrum aqueous extract of black cardamom, may have cognitive-enhancing potential in healthy working professionals aged 35–65 years. In this randomized, double-blind, placebo- and active-controlled 14-day trial, daily supplementation with 250 mg of MA2-24 was associated with improvements in attention, cognitive flexibility, processing speed, and executive function compared with placebo. These effects were observed from day 1 and were maintained through day 14, with performance generally comparable to that observed with caffeine across several cognitive outcomes. Co-administration of MA2-24 with caffeine appeared to extend the duration of cognitive performance benefits, with improvements observed through 5 h post-dose and selective effects persisting up to 8 h. Overall, these findings suggest that MA2-24 may represent a promising natural nootropic ingredient for functional food and nutraceutical applications. Nevertheless, larger, longer-duration, and mechanistic studies are needed to confirm these findings and further elucidate its mechanisms of action.

## Data Availability

The original contributions presented in the study are included in the article/Supplementary material, further inquiries can be directed to the corresponding author.
