# Supplementary material for: Influence of a full-spectrum aqueous extract of black cardamom (Amomum subulatum Roxb.) fruits on focus/alertness and executive function: a randomized, double-blind, placebo-and active- controlled, 14-day comparative study in healthy working professionals
Source: Front Neurosci. 2026 Sep 29;20:1879347. doi: 10.3389/fnins.2026.1879347 (PMC13662912; doi:10.3389/fnins.2026.1879347)
Supplement: Supplementary file 1 [file Supplementary_file_1.docx]

Supplementary data

**Table S1.** Inclusion and exclusion criteria

**Inclusion criteria**

1. Male and female working professionals between 35 and 65 years of age (inclusive).
2. Individuals with a BMI ranging from 18.5 to 29.9 kg/m² (inclusive). Participants must commit to maintaining their usual dietary and physical activity habits throughout the study duration.
3. Participants must have a Fatigue Severity Scale (FSS) score greater than 4 and be willing to maintain their regular lifestyle. They must also abstain from medications, alcohol, and caffeine before each study visit.
4. Participants must avoid vigorous physical activity for at least 12 hours prior to each study visit.
5. Female participants of reproductive age must agree to use medically approved contraceptive methods throughout the study and present a negative urine pregnancy test (UPT) at the screening visit.
6. Participants must demonstrate basic computer literacy, have prior experience with computerized testing, and provide signed informed consent.
7. Participants must be willing and able to follow all study requirements, take the investigational product as directed, attend all scheduled visits, comply with study restrictions, and complete the full study protocol.

**Exclusion criteria**

1. Individuals currently using any substances or medications that may enhance cognition, mood, or energy (including alcohol, tobacco, or other CNS-active agents).
2. Known hypersensitivity or previous allergic reactions to any component of the investigational product.
3. Individuals with moderate to severe fatigue or a clinical diagnosis of chronic fatigue syndrome.
4. Presence of malignant disease, end-stage organ failure, or laboratory abnormalities that, in the investigator's judgment, may increase risk or interfere with study evaluations.
5. Includes but is not limited to uncontrolled metabolic disorders (e.g., diabetes, thyroid disease), serious conditions such as cancer, renal failure, HIV, immunodeficiency, hepatic or biliary disorders, arthritis, or cardiac disease, as determined by the investigator.
6. Diagnosed psychiatric conditions other than anxiety or depression, sleep disorders, or use of medications for sleep.
7. Systolic blood pressure >160 mmHg or diastolic blood pressure >100 mmHg at the screening visit.
8. Use of any of the products like anxiolytics, antidepressants, antipsychotics, anticonvulsants, antihypertensives, centrally acting corticosteroids, opioid analgesics, hypnotics, or prescription sleep aids. Also excluded are individuals with a history (current or past) of alcohol abuse.
9. Female participants who are pregnant, breastfeeding, planning to become pregnant during the study period, or who test positive for pregnancy at screening or randomization.
10. Participation in another clinical trial involving investigational drugs or devices within 3 months prior to enrollment.

**Table S2**: Demographics and screening characteristics of participants in study groups.

| Characteristics | Caffeine | MA2-24 | MA2-24+ Caffeine | Placebo |
| --- | --- | --- | --- | --- |
| Gender (Male/Female) | 12/12 | 16/8 | 14/10 | 17/7 |
| Age (Years) | 43.08 ± 6.4 | 45.29 ± 7.14 | 42.79 ± 4.7 | 45.08 ± 6.3 |
| Height (cm) | 165.06 ± 8.1 | 166.90 ± 7.3 | 166.63 ± 8.7 | 166.71 ± 7.6 |
| Weight (kg) | 68.16 ± 8.1 | 68.27 ± 9.9 | 68.53 ± 12.2 | 68.65 ± 8.8 |
| BMI (kg/m^2^) | 25.02 ± 2.4 | 24.44 ± 2.6 | 24.51 ± 2.8 | 24.68 ± 2.6 |
| Temp (°F) | 98.12 ± 0.4 | 97.90 ± 0.5 | 98.0 ± 0.4 | 97.80 ± 0.5 |
| Heart rate (bpm) | 80.96 ± 11.0 | 80.58 ± 10.0 | 82.0 ± 9.2 | 81.46± 9.6 |
| Systolic BP (mm/Hg) | 119.58 ± 7.8 | 123.58 ± 10.6 | 118.38 ± 9.9 | 122.67 ± 9.5 |
| Diastolic BP (mm/Hg) | 78.54 ± 8.6 | 82.33 ± 8.0 | 79.42 ± 7.7 | 80.88 ± 8.0 |

**Table S3**. Baseline Vitals of participants in the study groups

| Characteristics | Temp (°F) | Heart rate (bpm) | Systolic BP (mm/Hg) | Diastolic BP (mm/Hg) |
| --- | --- | --- | --- | --- |
| Placebo |  |  |  |  |
| Baseline | 97.84 ± 0.5 | 84.83 ± 9.2 | 120.83 ± 8.8 | 78.08 ± 8.5 |
| 1 h | 98.21 ± 0.3 | 82.46 ± 5.3 | 119.67 ± 8.0 | 78.0 ± 6.0 |
| 3 h | 98.23 ± 0.2 | 77.88 ± 5.4 | 120.50 ± 5.6 | 76.88 ± 6.2 |
| 5 h | 97.99 ± 0.3 | 79.88 ± 4.0 | 120.75 ± 5.7 | 78.42 ± 6.6 |
| 8 h | 98.15 ± 0.4 | 79.17 ± 4.4 | 118.88 ± 5.9 | 75.29 ± 4.5 |
| MA2-24 |  |  |  |  |
| Baseline | 97.95 ± 0.4 | 83.25 ± 10.8 | 121.92 ± 8.1 | 79.25 ± 7.9 |
| 1 h | 98.29 ± 0.4 | 80.08 ± 6.3 | 120.42 ± 4.4 | 77.42 ± 6.0 |
| 3 h | 98.20 ± 0.4 | 77.58 ± 7.2 | 120.25 ± 6.3 | 75.75 ± 6.7 |
| 5 h | 98.05 ± 0.4 | 78.04 ± 5.8 | 120.13 ± 4.8 | 76.58 ± 5.6 |
| 8 h | 98.12 ± 0.3 | 77.79 ± 5.0 | 120.42 ± 5.4 | 77.38 ± 6.5 |
| Caffeine |  |  |  |  |
| Baseline | 97.83 ± 0.4 | 82.67 ± 11.5 | 122.75 ± 10.6 | 81.75 ± 6.8 |
| 1 h | 98.20 ± 0.4 | 78.63 ± 6.9 | 121.29 ± 7.1 | 80.21 ± 5.9 |
| 3 h | 98.20 ± 0.3 | 78.88 ± 6.4 | 120.83 ± 7.8 | 79.46 ± 6.4 |
| 5 h | 98.00 ± 0.3 | 79.25 ± 6.6 | 121.58 ± 7.0 | 77.83 ± 5.5 |
| 8 h | 98.15 ± 0.3 | 77.67 ± 5.4 | 120.75 ± 6.2 | 76.38 ± 6.8 |
| Caffeine+MA2-24 |  |  |  |  |
| Baseline | 97.89 ± 0.6 | 84.42 ± 8.1 | 119.58 ± 9.2 | 78.75 ± 8.7 |
| 1 h | 98.02 ± 0.3 | 81.75 ± 5.6 | 119.58 ± 6.0 | 78.25 ± 5.8 |
| 3 h | 98.04 ± 0.3 | 76.71 ± 4.9 | 119.46 ± 5.6 | 77.88 ± 5.5 |
| 5 h | 97.98 ± 0.4 | 80.42 ± 5.9 | 119.63 ± 6.0 | 77.96 ± 4.4 |
| 8 h | 98.06 ± 0.3 | 78.25 ± 5.3 | 120.21 ± 5.3 | 77.50 ± 5.7 |

**Table S4**. SAT-Correct Responses

| Group | Day 1 | Day7 | Day 14 |
| --- | --- | --- | --- |
| 1 hour | | |  |
| Placebo | 1.71 ± 5.17 | -1.60 ± 4.65 | 3.4 ± 8.55 |
| MA2-24 | 5.83 ± 3.97 | 4.57 ± 2.40 | 10.86 ± 7.72 |
| Caffeine | 7.63 ± 6.08 | 5.10 ± 4.96 | 15.77 ± 4.37 |
| MA2-24+Caffeine | 6.58 ± 7.53 | 7.10 ± 5.14 | 17.73 ± 6.18 |
| 3 hour | | | |
| Placebo | 1.96 ± 5.41 | -0.95 ± 6.06 | 2.95 ± 8.80 |
| MA2-24 | 7.46 ± 5.32 | 4.19 ± 2.60 | 11.24 ± 7.49 |
| Caffeine | 7 ± 7 | 4.38 ± 4.98 | 13.95 ± 5.54 |
| MA2-24+Caffeine | 9 ± 6.74 | 7.62 ± 5.89 | 20.91 ± 6.47 |
| 5 hour | | | |
| Placebo | 2.04 ± 3.93 | 0.15 ± 6.01 | 1.45 ± 10.96 |
| MA2-24 | 2.79 ± 5.33 | 3.05 ± 3.23 | 7 ± 5.22 |
| Caffeine | 1.71 ± 4.60 | 1.43 ± 4.52 | 3.32 ± 8.98 |
| MA2-24+Caffeine | 6.54 ± 4.86 | 4.48 ± 7.00 | 13.45 ± 5.47 |
| 8 hour | | | |
| Placebo | 2.08 ± 4.79 | -1.55 ± 6.55 | -4.3 ± 8.21 |
| MA2-24 | 3.83 ± 3.09 | -1.24 ± 2.57 | -3 ± 9.43 |
| Caffeine | 3.33 ± 2.93 | 0.95 ± 4.10 | -4.32 ± 7.11 |
| MA2-24+Caffeine | 3.46 ± 4.30 | 1.67 ± 5.81 | 7.55 ± 5.62 |

**Table S5**. SAT-Reaction Time

| Group | Day 1 | Day7 | Day 14 |
| --- | --- | --- | --- |
| 1 hour | | | |
| Placebo | -64.71 ± 45.69 | -21.55 ± 172.38 | -64.35 ± 202.30 |
| MA2-24 | -198.92 ± 113.46 | -182.81 ± 61.74 | -230.14 ± 218.79 |
| Caffeine | -258.33 ± 130.51 | -236.23 ± 72.25 | -256.91 ± 188.01 |
| MA2-24+Caffeine | -273.42 ± 155.50 | -226.23 ± 72.29 | -261.86 ± 208.27 |
| 3 hour | | | |
| Placebo | -38.04 ± 39.54 | 6.5 ± 136.58 | -39.55 ± 288.97 |
| MA2-24 | -162.96 ± 102.87 | -145.48 ± 72.68 | -315.33 ± 201.18 |
| Caffeine | -232.42 ± 100.68 | -218.09 ± 66.81 | -227.23 ± 263.21 |
| MA2-24+Caffeine | -210.21 ± 162.67 | -179.23 ± 91.11 | -201 ± 217.16 |
| 5 hour | | | |
| Placebo | 35.58 ± 46.04 | -15.45 ± 123.02 | 36.3 ± 214.89 |
| MA2-24 | -156.08 ± 97.06 | -69.95 ± 45.59 | -207.43 ± 255.78 |
| Caffeine | -58.50 ± 111.88 | -65.73 ± 58.75 | -58.32 ± 268.88 |
| MA2-24+Caffeine | -230.71 ± 151.74 | -61.27 ± 72.76 | -224.36 ± 229.32 |
| 8 hour | | | |
| Placebo | -45.33 ± 45.44 | 26.8 ± 112.95 | -41.65 ± 262.58 |
| MA2-24 | -66.63 ± 96.13 | -21.52 ± 71.58 | -67.71 ± 319.03 |
| Caffeine | -32.38 ± 145.94 | -26.41 ± 61.29 | -36.09 ± 228.65 |
| MA2-24+Caffeine | -132.46 ± 162.90 | -23.77 ± 47.65 | -129.45 ± 200.66 |

**Table S6**. SAT-Errors

| Group | Day 1 | Day7 | Day 14 |
| --- | --- | --- | --- |
| 1 hour | | | |
| Placebo | -0.79 ± 1.67 | -0.1 ± 2.45 | -0.75 ± 4.74 |
| MA2-24 | -2.54 ± 0.72 | -1.67 ± 2.08 | -2.52 ± 2.23 |
| Caffeine | -2.96 ± 0.81 | -2.59 ± 2.02 | -3.32 ± 2.90 |
| MA2-24+Caffeine | -3.75 ± 1.03 | -2.52 ± 1.94 | -2.95 ± 2.55 |
| 3 hour | | | |
| Placebo | -0.50 ± 2.28 | -0.55 ± 2.46 | 0.6 ± 4.92 |
| MA2-24 | -1.83 ± 0.92 | -2.14 ±2.69 | -3.38 ± 2.54 |
| Caffeine | -2.42 ± 0.78 | -2.18 ± 2.56 | -2.82 ± 4.09 |
| MA2-24+Caffeine | -3.46 ± 0.93 | -2.81 ± 1.89 | -2.73 ± 3.40 |
| 5 hour | | | |
| Placebo | 1.29 ± 2.63 | -1.1 ± 2.45 | 1.4 ± 5.11 |
| MA2-24 | -1.50 ± 0.88 | -1.71 ± 2.76 | -2.62 ± 2.75 |
| Caffeine | -1.96 ± 1.08 | -1.23 ± 2.65 | -1.23 ± 3.24 |
| MA2-24+Caffeine | -2.21 ± 0.88 | -2.29 ± 1.95 | -2.68 ± 2.34 |
| 8 hour | | | |
| Placebo | 0.46 ± 2.86 | 0.65 ± 2.48 | 0.95 ± 7.21 |
| MA2-24 | -1.04 ± 0.75 | -0.62 ± 2.25 | -2.71 ± 3.65 |
| Caffeine | -0.38 ± 0.97 | -0.32 ± 2.97 | -0.68 ± 3.34 |
| MA2-24+Caffeine | -1.71 ± 0.91 | -0.62 ± 1.99 | -2.55 ± 2.67 |

**Table S7**. SDC- Correct Responses

| Group | Day 1 | Day7 | Day 14 |
| --- | --- | --- | --- |
| 1 hour | | | |
| Placebo | 2.58 ± 6.47 | -0.6 ± 5.25 | -1.35 ± 4.17 |
| MA2-24 | 8.29 ± 9.68 | 3.76 ± 2.79 | 2.76 ± 2.26 |
| Caffeine | 10.13 ± 4.76 | 6.41 ± 3.50 | 7.27 ± 6.63 |
| MA2-24+Caffeine | 14.04 ± 4.38 | 8.09 ± 3.42 | 12.05 ± 5.92 |
| 3 hour | | | |
| Placebo | -1.29 ± 7.87 | 1.55 ± 4.59 | 0.25 ± 5.34 |
| MA2-24 | 6.50 ± 7.14 | 5.67 ± 3.60 | 6.76 ± 4.71 |
| Caffeine | 9.25 ± 4.61 | 7.32 ± 3.23 | 8.95 ± 8.21 |
| MA2-24+Caffeine | 11.79 ± 3.95 | 7.91 ± 3.77 | 11.77 ± 6.25 |
| 5 hour | | | |
| Placebo | -2.25 ± 6.26 | 1.35 ± 5.73 | -1.95 ± 5.42 |
| MA2-24 | 2 ± 9.70 | 3.57 ± 2.16 | 5.52 ± 2.73 |
| Caffeine | 1.13 ± 5.74 | 1.59 ± 4.20 | 3.18 ± 4.94 |
| MA2-24+Caffeine | 12 ± 4.19 | 4.73 ± 2.81 | 8.82 ± 3.89 |
| 8 hour | | | |
| Placebo | -1.13 ± 7.80 | -0.8 ± 4.01 | -2.55 ± 4.32 |
| MA2-24 | 0.46 ± 8.10 | 0.33 ± 4.60 | 1.86 ± 4.79 |
| Caffeine | -1.79 ± 3.90 | -2.41 ± 3.66 | -2.91 ± 5.75 |
| MA2-24+Caffeine | 1.33 ± 4.56 | 1 ± 2.67 | 4.68 ± 3.48 |

**Table S8**. SDC- Errors

| Group | Day 1 | Day7 | Day 14 |
| --- | --- | --- | --- |
| 1 hour | | | |
| Placebo | -0.13 ± 1.12 | -0.15 ± 1.39 | 0.05 ± 1.47 |
| MA2-24 | -0.92 ± 0.93 | -1.05 ± 0.86 | -1.4 ± 1.10 |
| Caffeine | -1.50 ± 0.72 | -1.45 ± 1.01 | -1.45 ± 0.96 |
| MA2-24+Caffeine | -1.54 ± 0.83 | -1.36 ± 1.09 | -1.82 ± 0.80 |
| 3 hour | | | |
| Placebo | 0.33 ± 0.96 | 0.55 ± 0.76 | -0.55 ± 2.01 |
| MA2-24 | -1.13 ± 0.80 | -1.14 ± 1.01 | -1.8 ± 1.36 |
| Caffeine | -1.67 ± 1.09 | -1.55 ± 0.96 | -1.95 ± 0.90 |
| MA2-24+Caffeine | -1.46 ± 0.93 | -2.09 ± 1.60 | -1.77 ± 1.07 |
| 5 hour | | | |
| Placebo | 0.08 ± 0.93 | -0.25 ± 1.74 | -0.4 ± 1.93 |
| MA2-24 | -0.29 ± 0.75 | -0.57 ± 0.81 | -1.9 ± 1.12 |
| Caffeine | -0.33 ± 0.76 | -0.41 ± 0.96 | -0.77 ± 1.48 |
| MA2-24+Caffeine | -0.75 ± 0.99 | -0.68 ± 0.95 | -1.64 ± 1.00 |
| 8 hour | | | |
| Placebo | 0.29 ± 1.04 | -0.2 ± 1.47 | 0.1 ± 1.62 |
| MA2-24 | -0.21 ± 0.78 | -0.24 ± 0.77 | -1.05 ± 1.00 |
| Caffeine | -0.13 ± 0.95 | -0.18 ± 0.91 | -0.68 ± 1.43 |
| MA2-24+Caffeine | 0.13 ± 0.95 | -0.45 ± 1.57 | -1.45 ± 0.80 |

**Table S9**. Simple -Stroop Reaction Time

| Group | Day 1 | Day7 | Day 14 |
| --- | --- | --- | --- |
| 1 hour | | | |
| Placebo | -30 ± 83.90 | -66.05 ± 111.71 | 14.55 ± 110.84 |
| MA2-24 | -83.54 ± 33.55 | -132.71 ± 46.87 | -115.95 ± 108.10 |
| Caffeine | -92 ± 33.57 | -170.27 ± 60.84 | -162.64 ± 71.23 |
| MA2-24+Caffeine | -142.29 ± 27.99 | -130.09 ± 75.11 | -181 ± 67.56 |
| 3 hour | | | |
| Placebo | -25 ± 72.16 | -24.2 ± 110.81 | -29.2 ± 90.14 |
| MA2-24 | -66.33 ± 23.51 | -190.24 ± 66.07 | -121.81 ± 90.22 |
| Caffeine | -79.79 ± 41.38 | -152.05 ± 61.72 | -133.36 ± 82.80 |
| MA2-24+Caffeine | -127.54 ± 30.32 | -143.27 ± 66.57 | -169.45 ± 74.14 |
| 5 hour | | | |
| Placebo | -40.54 ± 73.52 | -37.95 ± 103.30 | -30.2 ± 129.84 |
| MA2-24 | -48.17 ± 68.24 | -90.19 ± 84.14 | -119.24 ± 109.94 |
| Caffeine | -25.63 ± 56.09 | -78.5 ± 69.11 | -53.91 ± 64.51 |
| MA2-24+Caffeine | -107.75 ± 33.8 | -70.41 ± 54.76 | -117.23 ± 104.13 |
| 8 hour | | | |
| Placebo | 35.96 ± 65.89 | 13.9 ± 49.53 | 48.35 ± 115.26 |
| MA2-24 | 23 ± 51.84 | -14.86 ± 61.80 | 38.71 ± 56.40 |
| Caffeine | 30.25 ± 33.18 | 28.73 ± 42.41 | 59.5 ± 96.81 |
| MA2-24+Caffeine | 24.67 ± 96.32 | -8.32 ± 45.16 | 35.27 ± 80.35 |

**Table S10**. Stroop -Reaction Time

| Group | Day 1 | Day7 | Day 14 |
| --- | --- | --- | --- |
| 1 hour | | | |
| Placebo | 34.83 ± 87.67 | -26.15 ± 75.21 | 27.85 ± 144.86 |
| MA2-24 | -40.88 ± 20.16 | -128.33 ± 87.89 | -105.14 ± 99.53 |
| Caffeine | -74.54 ± 61.90 | -111.82 ± 63.45 | -127.09 ± 76.66 |
| MA2-24+Caffeine | -86.75 ± 82.85 | -131.27 ± 67.71 | -154.86 ± 102.47 |
| 3 hour | | | |
| Placebo | 24.38 ± 105.08 | 54.3 ± 69.19 | 52.1 ± 90.89 |
| MA2-24 | -34.54 ± 28.03 | -159.43 ± 56.74 | -176.05 ± 88.83 |
| Caffeine | -62.58 ± 23.31 | -124.86 ± 79.11 | -218.45 ± 115.02 |
| MA2-24+Caffeine | -67.67 ± 82.88 | -151.91 ± 64.95 | -251.09 ± 71.87 |
| 5 hour | | | |
| Placebo | -8.42 ± 94.47 | 55.65 ± 85.91 | 43.75 ± 132.77 |
| MA2-24 | -21.92 ± 20.30 | 34.19 ± 111.71 | -85.38 ± 84.44 |
| Caffeine | -15.88 ± 34.97 | 88.73 ± 91.31 | -19.41 ± 103.82 |
| MA2-24+Caffeine | -73.71 ± 107.25 | -71.32 ± 74.49 | -165 ± 130.06 |
| 8 hour | | | |
| Placebo | -15.88 ± 112.10 | 36.8 ± 89.21 | 36.95 ± 187.23 |
| MA2-24 | -9.04 ± 33.84 | 48.90 ± 126.10 | -34.38 ± 111.19 |
| Caffeine | -16.58 ± 29.38 | 90.18 ± 88.24 | 77.95 ± 197.59 |
| MA2-24+Caffeine | -4.21 ± 37.75 | 63.23 ± 102.24 | -23 ± 64.49 |
